# Experimentally quantifying anion polarizability at the air/water interface

**DOI:** 10.1038/s41467-018-03598-x

**Published:** 2018-04-03

**Authors:** Yujin Tong, Igor Ying Zhang, R. Kramer Campen

**Affiliations:** 1 Fritz Haber Institute of the Max Planck Society, 14195 Berlin, Germany; 2 Department of Chemistry, Fudan University, 200433 Shanghai, China

## Abstract

The adsorption of large, polarizable anions from aqueous solution on the air/water interface controls important atmospheric chemistry and is thought to resemble anion adsorption at hydrophobic interfaces generally. While the favourability of adsorption of such ions is clear, quantifying adsorption thermodynamics has proven challenging because it requires accurate description of the structure of the anion and its solvation shell at the interface. In principle anion polarizability offers a structural window, but to the best of our knowledge there has so far been no experimental technique that allowed its characterization with interfacial specificity. Here, we meet this challenge using interface-specific vibrational spectroscopy of Cl–O vibrations of the ${\mathrm{ClO}}_4^ -$ anion at the air/water interface and report that the interface breaks the symmetry of the anion, the anisotropy of ${\mathrm{ClO}}_4^ -$’s polarizability tensor is more than two times larger than in bulk water and concentration dependent, and concentration-dependent polarizability changes are consistent with correlated changes in surface tension.

## Abstract (web summary)

Understanding anion-specific interactions with hydrophobic interfaces is challenging due to an absence of local structural probes. Here, the authors experimentally quantify the anisotropy of perchlorate’s polarizability at the air/water interface, a window into anion and solvation shell structure.

## Introduction

The adsorption of anions on hydrophobic interfaces controls important chemistry on aerosol surfaces and determines the stability of proteins, colloids and foams in a wide variety of environmental, physiological and engineered settings. Anion adsorption on the air/water interface, the paradigmatic hydrophobic surface, has been particularly well studied^1–11^. Perhaps the simplest question one can ask of this system is the following: Is anion adsorption at the air/water interface thermodynamically favourable?

In principle measurements of surface tension of the air/water interface as a function of bulk ionic strength provide such insight. Decades of such measurements have confirmed that surface tensions of aqueous salt solutions increase with increasing ionic strength, those of acids decrease, and that the magnitude of the effect depends strongly on anion and only weakly on cation^1,12–16^. Historically the first observation was rationalized by Wagner, Onsager and Samaras (WOS) in their extension of the Debye–Hückel theory of bulk aqueous electrolytes to interfaces. Within this description anions are excluded from the air/water interface because exposure to a low dielectric phase leads to an enormous, unfavourable electrostatic self-energy^17,18^. While qualitatively consistent with surface tension measurements of salt solutions, this approach does not explain surface tension trends for acids or specific ion effects.

Motivated by reaction rates of gaseous species with solvated anions in atmospheric aerosols that were unexpectedly fast^19^, subsequent work—simulation using classical polarizable force fields, various surface sensitive spectroscopies^2,11,20,21^, dielectric continuum theory^22–25^ and properly parameterized fixed charge models^26^—has shown this Debye–Hückel inspired view to be incorrect. Large polarizable anions exist in higher concentrations at the air/water interface than in the adjoining bulk liquid. While the qualitative picture is clear, understanding of quantitative trends in anion adsorption, e.g. why does I^−^ adsorb more strongly than Cl^−^, and gaining atomically resolved insight into the driving force of anion adsorption has proven challenging.

In WOS theory ions are nonpolarizable point charges. As illustrated by Levin for an idealized anion, lack of polarizability induces a large, unfavourable, electrostatic self-energy in adsorbing anions: for a finite sized, ideal, polarizable anion essentially all charge density shifts towards the aqueous phase as the ion approaches the interface^23^. While it is thus clear ions must be polarizable to adsorb at the air/water interface, the contribution of ion polarization (i.e. the product of applied field and polarizability *p* = *α* ⋅ *E*) to the free energy of adsorption (Δ*G*_ads_) is not obvious. Initial studies describing ion adsorption in classical simulations concluded that explicit description of ion polarizability was critical and that the relative surface propensity of different ions was proportional to their polarizability and radius (i.e. large, soft, polarizable anions more favourably adsorb)^4,9^. However subsequent simulation studies have found that anion adsorption occurs in properly parameterized classical models without explicit description of polarizability, that relative anion polarization does not correlate with experimental and simulated trends in ion adsorption propensity, and that simulated interface active anions may be similarly polarized in bulk water and at the interface^21,26–30^.

Recent theoretical work in continuum models, and classical and ab-initio simulation has clarified that Δ*G*_ads_ of anion adsorption is dominated by a balance between large favourable cavitation and unfavourable desolvation energies: moving an anion from bulk to the air/water interface reduces the energetic cost to forming a cavity in water but also reduces the favourable interaction of the anion with, at least part of, its solvation shell^31–34^. The contribution of ion polarization to Δ*G*_ads_, the change in system free energy when adding a dipole to the interface, is relatively small. While accurately describing anion polarization thus appears to be relatively unimportant in decomposing anion Δ*G*_ads_, Pollard and Beck^34^ have highlighted that quantitative insight into Δ*G*_ads_ requires accurate description of the structure of the anion and its first solvation shell both in bulk and at the interface. Gaining such insight is a formidable theoretical challenge because the local electrical fields are as high as 1 V/Å.

Much theoretical progress could be made if there were experimental observables of the structure of the anion and its first solvation shell both in bulk and at the interface. This experimental challenge is particularly formidable because the structure of the anion and its solvation shell are correlated and both might be expected to change on moving from bulk to the interface. From the monoatomic anion’s perspective one might imagine structure changing because the ion electron density reflects the underlying asymmetry of electron density of the solvent, while for multiatomic anions interface induced changes in bond lengths and angles are additionally possible. From water’s perspective clearly moving to the interface must result in a change in structure of the solvation shell but the details of this change must be a function of changes in anion structure. Much prior work has shown that anion polarizability is exceptionally sensitive to both anion structure and that of its local solvation shell^35,36^. Thus while the contribution of ion polarization to Δ*G*_ads_ may be small, an experimental observable of anion polarizability in bulk and at the interface should offer an important constraint on anion and solvation shell structure.

The adsorption of the perchlorate anion at the air/water interface is known to be favourable^9^. In this work we experimentally quantify, using interface-specific vibrational spectroscopy, the increase in polarizability anisotropy of perchlorate on moving from bulk water to the interface and show that anisotropy increases with increasing perchlorate bulk concentration. Using a simple computational model we quantitatively relate the experimentally observed increase in polarizability anisotropy with increasing bulk concentration to increases in interfacial field (consistent with prior measurements of concentration-dependent surface potential^37^), ${\mathrm{ClO}}_4^ -$ dipole, and relative bond length of one Cl–O bond with respect to the other three with increasing concentrations of bulk HClO_4_. Quantitative theoretical insights into the driving force of anion adsorption at the air/water interface, and specific ion effects more generally, require accurate calculation of both the structure of ions and their first solvation shell at aqueous interfaces. Experimental measurements of anion polarizability offer a window into such structure unavailable by other means.

## Results

### Optically probing the polarizability anisotropy of perchlorate

The Raman depolarization ratio (*ρ*) is a useful means of probing anion polarizability in bulk H_2_O. Given an isotropic distribution of ions in liquid water and a molecular coordinate system (*a, b, c*) in which *a* (or *b*) is taken to be perpendicular to the net deformation of a normal mode and *c* parallel, one writes^38^

1 $$\rho = \frac{{{{I}}_ \bot }}{{{{I}}_{||}}} = \frac{3}{{4 + 5\left( {\frac{{1 + 2R}}{{R - 1}}} \right)^2}}$$

where *I*_⊥_ and *I*_||_ are the intensity of inelastic scattered light measured perpendicular and parallel to the plane polarized incident field and $R = \frac{{\partial \alpha _{aa}^{(1)}/\partial Q}}{{\partial \alpha _{cc}^{(1)}/\partial Q}}$. That is the Raman response of a particular mode can be quantitatively related to the change in the symmetry of the molecules polarizability tensor as the molecule is deformed in the mode’s characteristic manner. Given this definition of *ρ* is it perhaps unsurprising that several studies have shown that the ability to calculate the Raman response of modes that are strongly coupled to the environment is a sensitive test of the accuracy of polarizability models employed^39,40^. Because spontaneous Raman is not interface specific it is generally not possible to extract the *ρ* of interfacial anions. Clearly if we could, however, this observable could provide the sort of experimental constraint we seek.

Vibrationally resonant Sum Frequency (VSF) spectroscopy is a nonlinear optical, laser-based technique in which pulsed infrared (IR) and visible lasers are spatially and temporally overlapped at an interface and the output at the sum of the frequencies of the two incident beams monitored. The emitted VSF field is interface specific by its symmetry selection rules and a spectroscopy because as one tunes the frequency of one of the incident fields (in this case the IR) in resonance with an optically accessible transition the intensity of the emitted sum frequency field (*I*_sf_) increases by several orders of magnitude. Much prior work has shown that the intensity of the measured sum frequency response at a frequency *ω* is proportional to the change in polarizability (*α*_*ab*_) and dipole (*μ*_*c*_) with motion along the normal mode of frequency *ω*^41^:

2 $$\sqrt {{{I}}_{{\mathrm{sf}}}} \propto \chi _{ijk}^{(2)} \propto \beta _{abc}^{(2)} \propto - \frac{1}{{2\epsilon _0\omega }}\frac{{\partial \alpha _{ab}^{(1)}}}{{\partial Q}}\frac{{\partial \mu _c}}{{\partial Q}}$$

in which $\chi _{ijk}^{(2)}$ is the macroscopic nonlinear susceptibility in the lab coordinate system (*ijk*), $\beta _{abc}^{(2)}$ the molecular hyperpolarizability and both are third rank tensors. Because by varying experimental conditions, i.e. beam incident angles and field polarizations, one can selectively probe different components of *β*^(2)^, a correctly chosen ratio of intensities allows the direct measurement of $R = \frac{{\partial \alpha _{aa}^{(1)}/\partial Q}}{{\partial \alpha _{cc}^{(1)}/\partial Q}}$, and thus the possibility of extracting the Raman depolarization ratio of anions with interfacial specificity. That is, by comparing measurements of *ρ* for an anion in solution and at the air/water interface experimental estimates of anion polarizability anisotropy at aqueous interfaces (and the change in anion polarizability anisotropy on moving from bulk liquid water to the aqueous interface) are possible.

### VSF spectra of interfacial ${\mathrm{ClO}}_4^ -$

**Fig. 1:**
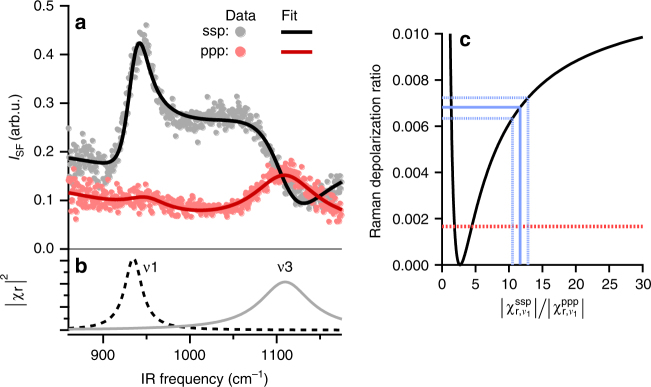
The spectral response of interfacial ${\mathrm{ClO}}_4^ -$. **a** SFG spectrum of 0.6 M HClO_4_ solution at the air/water interface measured under ssp (black traces) and ppp (red traces) polarization combinations. Circular symbols are the experimental observations; solid traces are the fit to Eq. (3). **b** Two components obtained from spectral fit, assigned to *ν*_1_ (A1 symmetry) and *ν*_3_ (F2 symmetry) vibrational modes of perchlorate respectively. **c** Calculated Raman depolarization ratio (black solid line) as a function of the measured VSF peak amplitude ratio ($\chi _{{\mathrm{r,}}\nu _1}^{{\mathrm{ssp}}}/\chi _{{\mathrm{r,}}\nu _1}^{{\mathrm{ppp}}}$); the blue solid line is the result of fitting the data shown in **a**, blue dashed lines are the estimated uncertainty in the amplitude ratio and the resulting Raman depolarization ratio and the red dashed line indicates the bulk value reported by prior authors [43]. The uncertainty in the Raman depolarization ratio is dominated by uncertainty in the fit of the observed spectra, see Supplementary Note 1 for details of calculation

Figure 1a shows the VSF spectra from the air/0.6 M HClO_4_ solution interface measured under the ssp (*s*-polarized SF, *s*-polarized visible, and *p*-polarized IR) (black circles) and ppp (red squares) polarization combinations. There are two resonances apparent in this frequency range. Fitting both spectra simultaneously with the Lorentzian lineshape model described in the Methods section results in resonances centred at 935 and 1110 cm^−1^ (Fig. 1b dotted lines, see Supplementary Note 1 for details of the data analysis).

Because both spectral features are absent in pure water, and are spectrally separated from resonances of water or likely impurities, they can be straightforwardly assigned by reference to Raman and IR measurements of bulk aqueous perchloric acid and perchlorate salt solutions^42–44^. In brief, the ${\mathrm{ClO}}_4^ -$ anion has four normal modes apparent in calculation: the *ν*_1_ at 930, the *ν*_2_ at 450, the *ν*_3_ at 1100 and the *ν*_4_ at 620 cm^−1^. All four are Raman active at all concentrations in bulk aqueous solution but the *ν*_1_ and *ν*_2_ are only apparent in IR absorption spectra at bulk concentrations greater than ≈11 M. This observation is a straightforward consequence of anion symmetry: at bulk concentrations below 11 M the ${\mathrm{ClO}}_4^ -$ anion has T_*d*_ symmetry (under which condition *ν*_1_ and *ν*_2_ are IR inactive) and at sufficiently high concentrations this symmetry is broken: either by ion pairing or, in the case of HClO_4_, by the presence of molecular acid. If we assign the resonance apparent in Fig. 1b at 935 cm^−1^ to the *ν*_1_ mode and that apparent at 1110 cm^−1^ to the *ν*_3_, we are left with an apparent incongruity. As shown in Eq. (2), VSF activity requires that a mode must be both IR and Raman active. This implies the ${\mathrm{ClO}}_4^ -$ anion must lose its T_*d*_ symmetry at the air/water interface at concentrations that are at least 15× lower than those at which T_*d*_ symmetry is lifted in bulk.

### Understanding why T_*d*_ symmetry is lifted in interfacial ${\mathrm{ClO}}_4^ -$

We imagine three possible mechanisms for the loss of T_*d*_ symmetry: consistent with recent work on other strong acids, molecular HClO_4_ may exist at the air/water interface at concentrations dramatically lower than in bulk^45^, ${\mathrm{ClO}}_4^ -$ may no longer have tetrahedral symmetry due to ion pairing, or it may not have tetrahedral symmetry due to, more general, solvation anisotropy at the interface. We tested the first possibility by collecting spectra from 0.6 M solutions of NaClO_4_. For this a similarly intense *ν*_1_ feature is observed, suggesting the likely cause of T_*d*_ symmetry lifting is not molecular acid (see Supplementary Note 2 for data).

To evaluate the possibility of T_*d*_ symmetry lifting due to ion pairing, it is necessary to understand how ion pairing might be expected to influence the *ν*_1_ and *ν*_3_ spectral response. In bulk solutions of perchlorate salts at concentrations above 1 M the centre frequency of perchlorate’s *ν*_3_ mode has been observed to continuously shift as a function of concentration^46^. This concentration-dependent spectral evolution has been assigned to the formation of weak, solvent-separated ion pairs. As mentioned above, in this concentration range the *ν*_1_ mode is infrared inactive. At still higher concentrations in bulk water, >11 M, perchlorate’s degenerate modes, i.e. *ν*_2_, *ν*_3_ and *ν*_4_, have been observed to split due to contact ion pair formation, where the degree of splitting is a function of the extent to which symmetry is broken^46,47^.

**Fig. 2:**
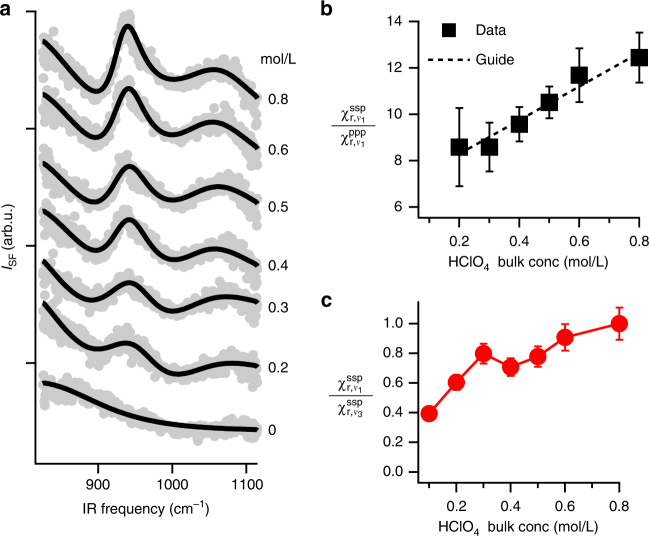
The concentration-dependent spectral response of interfacial ${\mathrm{ClO}}_4^ -$. **a** VSF spectra as a function of ${\mathrm{ClO}}_4^ -$ concentration below 1 M for the ssp polarization combinations. Grey dots are data and solid black lines the corresponding fits. **b** $\chi _{{\mathrm{r}},\nu _1}^{{\mathrm{ssp}}}/\chi _{{\mathrm{r}},\nu _1}^{{\mathrm{ppp}}}$ for the data in **a** and the SI illustrating the linearity of this ratio with respect to bulk HClO_4_ concentration (dotted line is a guide to the eye). The uncertainty at each point is extracted from the fits to the data as described in Supplementary Note 1. **c** $\chi _{{\mathrm{r}},\nu _1}^{{\mathrm{ssp}}}/\chi _{{\mathrm{r}},\nu _3}^{{\mathrm{ssp}}}$ extracted from the data in **a** and the SI. This ratio varies by ≈50% with change in bulk concentration of HClO_4_. As we show in Supplementary Note 3 and discuss in the text, changes of this magnitude imply a, concentration dependent, change in interfacial ${\mathrm{ClO}}_4^ -$’s orientation of less than 5°: the orientation of interfacial ${\mathrm{ClO}}_4^ -$ is concentration independent

As is discussed in detail below (see Fig. 2 for data) at bulk concentrations lower than 1 M HClO_4_ the *ν*_1_ mode is clearly VSF (and thus IR) active, the *ν*_3_ spectral response is quantitatively reproduced with a single centre frequency and line width: splitting or frequency shift of the *ν*_3_ resonance is not required to describe our data. We therefore conclude that neither contact nor solvent-separated ion pair formation explains the lifting of T_*d*_ symmetry. Given that VSF spectra collected at bulk concentrations below 1 M HClO_4_ are consistent with formation of neither weak, solvent-separated ion pairs nor contact ion pairs, we conclude that the lifting of T_*d*_ symmetry for interfacial ${\mathrm{ClO}}_4^ -$ (and thus the IR and VSF activity of the *ν*_1_ mode) must be the result of the intrinsic anisotropy of the solvation environment at the air/water interface: solvation anisotropy must induce sufficient structural deformation in the ${\mathrm{ClO}}_4^ -$ anion to lift the bulk T_*d*_ symmetry and make the *ν*_1_ mode IR, and VSF, active.

While this qualitative observation of a consequence of structural deformation is important, to make clear connections to theory it would be useful to quantify this deformation, and the resulting change in the perchlorate polarizability tensor. Given the *ν*_1_ spectral amplitudes extracted from the fit of the ssp and ppp data in Fig. 1a (see Supplementary Note 1 for full details of the fit), and assuming the ${\mathrm{ClO}}_4^ -$ anion is oriented such that one Cl–O points along the surface normal and that the anion has C_3*ν*_ symmetry, we can calculate the Raman depolarization ratio, i.e.* ρ*, for perchlorate ions at the interface (see Supplementary Note 3 for a description of the theory connecting measured *I*_sf_ to the Raman depolarization ratio). The result of this calculation is shown by the solid black line in Fig. 1c. The ratio of spectral amplitudes of the *ν*_1_ ssp and ppp shown in Fig. 1a suggests a *ν*_1_ *ρ* of 0.0068 ± 0.0005 (the uncertainty originates from both the average of three measurements and the spectral fit, see Supplementary Tables 1–3 for all results), or more than 2× larger than the same quantity for ${\mathrm{ClO}}_4^ -$ in bulk.

It is worth emphasizing that the result that interfacial ${\mathrm{ClO}}_4^ -$’s Raman depolarization ratio is significantly larger than bulk is insensitive to the simplifying assumptions required in its calculation. As we show in Supplementary Note 4, assuming the ${\mathrm{ClO}}_4^ -$ is oriented with one Cl–O bond at an increasing, nonzero angle with respect to the surface normal leads to slightly larger estimates for the depolarization ratio of *ν*_1_ while assuming the symmetry of the ${\mathrm{ClO}}_4^ -$ anion has decreased to C_2*ν*_ or C_∞*ν*_ leads to quantitatively similar results. Note also that because we measure the intensity of the emitted sum frequency light, and not the field, our measurements are equally consistent with one Cl–O bond along the surface normal and the remaining three oxygens pointing either towards the bulk liquid or air. Prior theoretical studies imply, at least in the low concentration limit, that the latter configuration is favoured^7,48,49^.

As noted above, changes in polarizability must be correlated with changes in anion nuclear structure. As we show in detail in Supplementary Note 5, a simple computational model suggests a *ρ* of 0.0068 is consistent with a 3% change in Cl–O bond length (for the Cl–O along the surface normal), and a permanent dipole moment of interfacial ${\mathrm{ClO}}_4^ -$ of 0.75 Debye (n.b. consistent with the absence of IR active *ν*_1_ and *ν*_2_ modes for the ${\mathrm{ClO}}_4^ -$ anion in vacuum or in bulk liquid water, perchlorate’s dipole moment in either bulk phase is below our detection limit). In this manner our experimental observable directly constrains the anisotropy of interfacial perchlorate’s polarizability tensor and places quantitative constraints on interfacial ${\mathrm{ClO}}_4^ -$ polarization at the air/water interface.

### Change in spectral response of interfacial ${\mathrm{ClO}}_4^ -$ with changing bulk concentration

To gain more insight into the fate of the ${\mathrm{ClO}}_4^ -$ anion at the air/water interface, we next measured its *ν*_1_ and *ν*_3_ spectral amplitudes as a function of bulk concentration of HClO_4_ from 0.1 to 0.8 M (higher concentrations lead to qualitative change in the spectral response, possibly the result of interface induced ion pairing, as shown in Supplementary Note 6). The concentration-dependent spectra collected under the ssp polarization condition are shown in Fig. 2a, the concentration-dependent ppp are plotted in Supplementary Fig. 1. Global fitting of both sets of data allows the extraction of the $\chi _{{\mathrm{r,}}\nu _1}^{{\mathrm{ssp}}}/\chi _{{\mathrm{r,}}\nu _1}^{{\mathrm{ppp}}}$ ratio as a function of bulk concentration. As shown in Fig. 2b changing bulk concentrations of HClO_4_ from 0.1 to 0.8 M leads to a change of this ratio from 8.58 ± 1.7 to 12.4 ± 1.1.

Because the VSF spectral response reports on the properties of interfacial ${\mathrm{ClO}}_4^ -$, it would clearly be useful if we could quantify the increase in interfacial concentration with increasing bulk concentration of HClO_4_. As we discuss in detail in Supplementary Note 7, accurately determining the interfacial concentration of anions at the interface is extremely challenging: inferring interfacial concentration from changes in surface tension with bulk concentration requires a simplifying model whose assumptions are difficult to independently evaluate, inferring interfacial population from the VSF or SHG spectral response requires assuming a concentration-independent molecular response that our results suggest is unlikely in the case of ${\mathrm{ClO}}_4^ -$ (discussed in more detail below) and, possibly, inferring interfacial concentration in x-ray photoemission measurements requires a reference for the inelastic mean free path of the electron that does not obviously exist^50^. Despite these limitations we have quantified the interfacial ${\mathrm{ClO}}_4^ -$ using surface tension and VSF measurements. The surface tension data suggest that increasing bulk concentrations from 0.1 → 1 M lead to an increase of the surface excess of ${\mathrm{ClO}}_4^ -$ from 0.3 × 10^−6^ → 1.1 × 10^−6^ mol/m^2^ while the VSF response suggests that over this concentration range the surface coverage increases from 0.5 → 1.0 monolayers.

Despite the uncertainty in interfacial ${\mathrm{ClO}}_4^ -$ concentration, reference to Fig. 1 makes clear that this change $\chi _{{\mathrm{r,}}\nu _1}^{{\mathrm{ssp}}}/\chi _{{\mathrm{r,}}\nu _1}^{{\mathrm{ppp}}}$ ratio from 8 → 13 implies an increase in the Raman depolarization ratio of *ν*_1_ of 0.0053 ± 0.0012 to 0.0071 ± 0.0004 (all depolarization ratios with their uncertainties are tabulated in Supplementary Table 3) over the same concentration range. Evidently, with increasing interfacial population, ${\mathrm{ClO}}_4^ -$ polarizability grows increasingly anisotropic. Using the same simple computational model discussed above, increasing the depolarization ratio from 0.005 to 0.007 is consistent with an increase in interfacial field from 139 to 171 (meV), an elongation in the Cl–O bond along the surface normal of 2.6–3.3% and a change in ${\mathrm{ClO}}_4^ -$ dipole moment from 0.6 to 0.76 Debye.

The relationship between $\chi _{{\mathrm{r,}}\nu _1}^{{\mathrm{ssp}}}/\chi _{{\mathrm{r,}}\nu _1}^{{\mathrm{ppp}}}$ and Raman depolarization ratio shown in Fig. 1 assumes that the ${\mathrm{ClO}}_4^ -$ is orientated such that one Cl–O group points along the surface normal. Applying this analysis to the data shown in Fig. 2a implicitly assumes that this orientation is concentration independent. Because the *ν*_1_ and *ν*_3_ normal modes are orthogonal, we would expect any concentration-dependent change in the orientation of interfacial ${\mathrm{ClO}}_4^ -$ to result in significant change in the $\chi _{{\mathrm{r,}}\nu _1}^{{\mathrm{ssp}}}/\chi _{{\mathrm{r,}}\nu _3}^{{\mathrm{ssp}}}$. As is shown in Fig. 2c the change we observe in this ratio as a function of bulk concentration is <50%. A change of this size is consistent with a, concentration dependent, change in the orientation of <5° (see Supplementary Note 4 for details of the calculation). We thus conclude that the orientation of interfacial ${\mathrm{ClO}}_4^ -$ is, to within the limits of our sensitivity, over 0.1–0.8 M range in bulk concentration, concentration independent.

Our results suggest the following model for ${\mathrm{ClO}}_4^ -$ at the air/water interface: on adsorption ${\mathrm{ClO}}_4^ -$ is polarized, i.e. it has a nonzero dipole moment, and the polarizability anisotropy changes due to a change in the bond length of the Cl–O that points along the surface normal relative to the three other Cl–O bonds. With increasing interfacial concentrations of ${\mathrm{ClO}}_4^ -$ the interfacial field increases, ion polarization increases (the dipole continues to grow) and the polarizability anisotropy continues to increase.

## Discussion

Modern dielectric continuum descriptions largely reproduce experimentally measured changes in surface tension with increasing concentrations of both acids and salts^24,31^. Notably the largest disagreements between experiment and theory, at least within the model of Levin and co-workers in which ions are approximated as hard spheres with fixed, concentration-independent radii, exist for ${\mathrm{ClO}}_4^ -$ solutions (both acids and salts). Levin and co-workers^51,52^ have suggested that this is likely the result of inaccuracies in the estimates of ionic radii for ${\mathrm{ClO}}_4^ -$. Our results are consistent with an alternative scenario in which anion polarizability (and ${\mathrm{ClO}}_4^ -$ radius) is interfacial concentration dependent. While our results imply the relationship between ${\mathrm{ClO}}_4^ -$ radius and interfacial concentration is monotonic, larger multivalent ions might be expected to have a more complicated interplay between structure, interfacial concentration and interfacial potential, all of which might be observed through the window of anion polarizability.

Atomistic simulation studies, whether employing classical or ab-initio potential energy surfaces, have largely reported either potentials of mean force for ion adsorption in the limit of infinite dilution or brute force simulations at a fixed ion concentration. As alluded to above, while important and informative these studies suffer from several possible shortcomings. The lack of experimental constraints on polarizability mean that estimates of polarizability from ab-initio simulations, and the local structure that produces them, cannot be validated and that classical polarizability models (and the manner in which they relate to local structure) cannot be parameterized. To further heighten the challenge, the surface potential of pure water is both difficult to measure experimentally and the subject of significant disagreement (by more than 0.5 V) in simulation treatments^53^. Thus one might expect that inaccuracies in surface potential of the pure water/air interface might, plausibly, compensate for inaccuracies in the description of local structure (and thus polarizability). Experiments of the sort described in this study gives a clear path forward through these challenges. Given experimental constraints on interfacial anion polarizability of the sort described in this study, descriptions of local anion structure inferred from ab-initio simulation can be validated and empirical polarizability models more accurately parameterized. Given a validated description of local ion structure systematically reducing errors in the calculation of (ion concentration dependent) surface potential is now much more straightforward.

Prior workers have performed studies similar in spirit to those shown here. Miyame et al.^6^ characterized the S–O stretch vibrations of ${\mathrm{SO}}_4^{2 - }$, while Motschmann and co-workers^10^ characterized the CN stretch vibrations of the potassium ferricyanide ion, i.e. ${\mathrm{Fe}}\left( {{\mathrm{CN}}} \right)_6^{4 - }$, as a function of bulk concentration at the air/water interface. Consistent with both calculation and other experimental approaches that suggest ${\mathrm{SO}}_4^{2 - }$ retains its bulk solvation shell at the air/water interface^4,54^, Miyame, Morita and Ouchi find interfacial ${\mathrm{SO}}_4^{2 - }$ to be essentially the same as bulk. In contrast Motschmann and co-workers found that CN modes that were IR inactive in bulk solution were apparent in the VSF spectrum, i.e. the interface induces a change in ferricyanide symmetry as it does for perchlorate. However, presumably because of the more structurally complicated anion, they were unable to quantify the resulting change in the polarizability tensor.

As is clear from Eq. (2), if a molecules hyperpolarizability and orientation are concentration independent one can extract a measurement of anion interfacial density as a function of bulk concentration by plotting the square root of the measured SF signal vs. bulk concentration. In a series of studies employing electronically resonant second harmonic measurements of a variety of anions at air/water interface, Saykally and co-workers^5,21^ have treated anion hyperpolarizability (and orientation) as concentration independent, fit adsorption isotherms to measurements of SHG signal as a function of bulk concentration, and calculated anion adsorption energies. Our results suggest that this type of data needs to be revisited. Because deformation of the perchlorate leads to an increase in transition dipole and polarizability, it is clear that given a VSF spectra collected under the ssp polarization condition, using this approach would overestimate adsorption energies (i.e. the molecular response of the perchlorate anion would increase with increasing concentration).

In summary, in the current study we have employed VSF spectroscopy and a simple computational model to study the behaviour of ${\mathrm{ClO}}_4^ -$ at the air/HClO_4_ solution interface. Consistent with much prior work our observations clearly demonstrate that ${\mathrm{ClO}}_4^ -$ is a surface active anion. We significantly extend these prior efforts by demonstrating that the presence of the interface induces deformation of the anion that causes a bulk forbidden mode to be VSF active due to change in anion symmetry, creates a nonzero dipole moment and leads to a change in the measured polarizability anisotropy. Our results suggest that increasing density of ${\mathrm{ClO}}_4^ -$ at the interface leads to an increasing interfacial field that increases ${\mathrm{ClO}}_4^ -$ polarization (i.e. increases ${\mathrm{ClO}}_4^ -$ dipole moment), increases ${\mathrm{ClO}}_4^ -$ structural deformation and makes the polarizability of ${\mathrm{ClO}}_4^ -$ increasingly anisotropic^4,5,9^. Extension of the approach we describe here should allow the possibility of directly quantifying the elements of ${\mathrm{ClO}}_4^ -$ polarizability tensor, rather than just their ratio, and enable a window onto local interfacial anion structure (particularly ion/ion correlation) and the possibility of reliably experimentally quantifying the thermodynamic importance of anion polarization.

The close connection we describe here between the dipole moment, structure and polarizability of interfacial anions with increasing interfacial field has not, to our knowledge, been previously considered but should be a quite general feature of anion, particularly polyvalent anion, adsorption at hydrophobic interfaces. As such its quantitative reproduction is a prerequisite for simulation approaches that attempt to offer microscopic insight into this phenomena.

## Methods

### Solution preparation

HClO_4_ (Suprapur, 70%, Merck) and NaClO_4_ (>99.99%, Sigma-Aldrich) were used as received. Solutions with the indicated concentrations were prepared by diluting the high concentration of HClO_4_ and NaClO_4_ in ultrapure H_2_O (18.3 MΩ cm; Milli-Q, Millipore). All solutions are prepared freshly before each measurement to limit degradation or contamination. VSF measurements in the C–H stretching and C=O region were employed to judge the quality of the solutions.

### Surface tension measurement

The concentration-dependent surface tensions were measured with a surface tensiometer (K12/K14 KRÜSS GmbH) at room temperature (25 ± 1 °C). A Du Noüy ring method with a chemically inert Pt ring was utilized. The sample volume was 50 mL. Each measured surface tension is an average of 20 separate measurements, collected by the Pt ring in and out of the interface without tearing the lamella.

### VSF measurement and spectral modelling

The VSF spectrometer employed for the current measurement, and in particular its power at long infrared wavelengths, has been described in detail in our previous studies^55,56^. In the interest of brevity only a brief description that pertinent to this measurement will be given here. The IR beam was generated from a commercial optical parametric amplifier (HE-TOPAS, Light Conversion) with a difference frequency generation (DFG) module. The full width half maximum (FWHM) of the beam at frequency region between 600 and 1200 cm^−1^ is typically 300 cm^−1^ with GaSe used as the DFG crystal. To probe the interfacial Cl–O stretch modes the centre frequency of the beam was tuned to ≈1000 cm^−1^. A narrow-band visible (VIS) pulse was produced from a home-made spectral shaper^55^. The beam is centred at 800 nm with a bandwidth of 15 cm^−1^. The energy per pulse of the IR and VIS at the sample surface was 5.8 and 15.4 μJ respectively. polarizations and energies of the incident fields at the interface were controlled using *λ*/2 plate, polarizer, *λ*/2 plate combinations. The two beams propagate in a coplanar fashion and focused on the samples using lenses with focal lengths of 10 and 25 cm and incident angles of 39.5 ± 0.5° and 65 ± 0.5° for the IR and VIS. All measurements were conducted in ambient conditions at room temperature and under the ssp (*s*-polarized SF, *s*-polarized visible, and *p*- polarized IR where *p* indicates polarization in the plane of incidence and *s* polarization orthogonal) and ppp polarization condition. Non-resonant signals from a gold thin film were used to correct for the frequency dependent IR intensity. The acquisition time for spectra of the gold reference and samples were 30 and 300 s, respectively.

To quantify the observed VSF spectral response, we adopted a Lorentzian lineshape model described and justified in much previous work by us and others^55–59^.

3 $${{I}}_{{\mathrm{sf}}}\left( {\omega _{{\mathrm{sf}}}} \right) \propto \left| {\chi _{{\mathrm{eff}}}^{(2)}} \right|^2 \propto \left| {\left| {\chi _{{\mathrm{nr}}}} \right|{\mathrm e}^{i\epsilon } + \mathop {\sum}\limits_n \frac{{\chi _{{\mathrm{r}},n}}}{{\omega _{{\mathrm{ir}}} - \omega _n + i{\Gamma }_n}}} \right|^2$$

where *I*_sf_(*ω*_sf_) is the normalized VSF intensity, $\chi _{{\mathrm{eff}}}^{(2)}$ is the effective second-order susceptibility, which depends on the experimental geometry, molecular hyperpolarizability and orientation. $\left| {\chi _{{\mathrm{nr}}}} \right|$ and $\epsilon$ are the non-resonant amplitude and phase and *χ*_r,*n*_, *ω*_*n*_ and *Γ*_*n*_ are the complex amplitude, centre frequency and line width of the *n*th resonance.

To actually analyse the data we fit the measured VSF spectrum using the Levenberg–Marquardt algorithm as implemented in the commercial visualization and analysis programme Igor Pro (Wavemetrics). Fitting spectra collected at each bulk concentration and polarization with this lineshape model results in an underdetermined minimization problem. Because bulk studies suggest that the centre frequencies and spectral shape of ${\mathrm{ClO}}_4^ -$ solution are concentration independent, we addressed these data by assuming that all spectra collected under a bulk concentration of HClO_4_ could be described with two resonances, each with a concentration independent line width, centre frequency and phase, and a non-resonant amplitude and phase that are also concentration independent. We accounted for the libration tail (only important in the ssp spectra) by assuming the libration has the centre frequency and line width from our previous study^56^. Details of the analysis, and all the parameters resulting from the fit, are given in Supplementary Note 1.

Recent work has shown that, at sufficiently charged interfaces, it is possible that the interfacial *χ*^(2)^ signal of specifies also present in the adjoining bulk aqueous phase, may be distorted by a *χ*^(3)^ response from oscillators within the electrical double layer. Such mixing is unlikely to effect the *ν*_1_ Cl–O spectral response (this mode is symmetry inactive away from the interface and thus has no spectral response within the double layer) and unlikely to effect the VSF spectra of either the *ν*_1_ or *ν*_3_ at the relatively low interfacial charge densities but high electrolyte concentration we explore here^60–62^.

### Data availability

All data is available from the corresponding author on request.

## Electronic supplementary material

Supplementary Information(PDF 1258 kb)
