# Supplementary material for: Experimentally quantifying anion polarizability at the air/water interface
Source: Nat Commun. 2018 Apr 3;9:1313. doi: 10.1038/s41467-018-03598-x (PMC5882839; doi:10.1038/s41467-018-03598-x)
Supplement: Supplementary file 1 — Supplementary Information(PDF 1258 kb) [file 41467_2018_3598_MOESM1_ESM.pdf]

Supplementary Note 1. Line Shape Analysis Details & Full Results

We do a line shape analysis of our experimental data by performing a global fit of spectra – using the Levenberg-Marquardt algorithm as implemented in the commercial graphing and analysis program Igor Pro (Wavemetrics) – collected under the *ssp* and *ppp* polarisation conditions for all bulk $HClO_4$ concentrations using the line shape expression described in the text. To quantitatively account for the effect of the, finite, visible pulse spectral width we additional convolve this response with a gaussian of width $\Delta\nu_{800}$. The spectrum of the visible pulse is independently measured before each VSF measurement. To do the fit we assume a libration (of interfacial water) whose center frequency and line width we have determined in our previous study [1] and further assume, for concentrations lower than 1 M $HClO_4$, that the center frequencies and damping constants of the ν_1 and ν_3 modes; the libration amplitude; and the nonresonant amplitude and phase are all independent of concentration. Uncertainties reported with each fit parameter (the estimated standard deviation in each paramters) are calculated from a linearization of the model, with respect to its parameters, near the best fit. The averages of parameters resulting from fits to three independent data sets are shown in Tables 1, 2, and 3. Two additional notes are worth adding. Firstly, because the 0.1 mol/L spectral response is particularly weak when measured under either *ssp* or *ppp* we found the $\chi^{ppp}_{\nu_1}$ to be particularly uncertain, *i.e.* 0.04 ± 0.14, we did not consider the 0.1 mol/L sample in any of the polarisability analysis (and this data was not shown in the Figure 2a in the manuscript or in Supplementary Figure 1 below). Secondly, we assume in the global fit that the amplitude of the ClO_4^- resonances is zero in the sample of pure water and fix them.

$[HClO_4]$(mol/L)	**0**	**0.1**	**0.2**	**0.3**	**0.4**	**0.5**	**0.6**	**0.8**
χ_{nr}					0.030 ± 0.002			
ϵ(rad)					0.731 ± 0.085			
$\Delta\nu_{800}$(cm^{-1})					12.2			
χ_{ν_1}	0	1.097 ± 0.106	1.872 ± 0.126	2.306 ± 0.139	2.418 ± 0.155	2.597 ± 0.171	2.780 ± 0.190	3.063 ± 0.239
$\tilde{\nu}_{\nu_1}$(cm^{-1})					935 ± 1.0			
Γ_{ν_1}(cm^{-1})					12.1 ± 0.7			
χ_{ν_3}	0	-9.335 ± 0.487	-10.389 ± 0.541	-9.683 ± 0.570	-11.461 ± 0.628	-11.190 ± 0.651	-10.265 ± 0.728	-10.293 ± 0.777
$\tilde{\nu}_{\nu_3}$(cm^{-1})					1110 ± 1.4			
Γ_{ν_3}(cm^{-1})					75.7 ± 1.2			
χ_{lib}					6.498 ± 0.242			
$\tilde{\nu}_{lib}$(cm^{-1})					832			
$\Gamma_{lib\nu_3}$(cm^{-1})					135			

Supplementary Table 1. Results of fits to data collected employing the *ssp* polarisation condition. The parameters shown in this and Table 2 are the result of a global fit to *ppp* and *ssp* spectra at all concentrations of $HClO_4$. Note that the spectral width of the 800 nm pulse was independently measured before each VSF measurement and the libration center frequency and line width were extracted from our previous work[1].

$[HClO_4]$(mol/L)	**0**	**0.1**	**0.2**	**0.3**	**0.4**	**0.5**	**0.6**	**0.8**
χ_{nr}					0.228 ± 0.004			
ϵ(rad)					4.53 ± 0.039			
$\Delta\nu_{800}$(cm^{-1})					12.2			
χ_{ν_1}	0	0.04 ± 0.14	0.218 ± 0.081	0.268 ± 0.075	0.253 ± 0.068	0.247 ± 0.065	0.238 ± 0.067	0.246 ± 0.059
$\tilde{\nu}_{\nu_1}$(cm^{-1})					935 ± 2.3			
Γ_{ν_1}(cm^{-1})					12.1 ± 0.6			
χ_{ν_3}	0	-2.699 ± 0.193	-2.370 ± 0.254	-5.523 ± 0.185	-5.441 ± 0.178	-6.978 ± 0.237	-6.205 ± 0.155	-6.124 ± 0.134
$\tilde{\nu}_{\nu_3}$(cm^{-1})					1110 ± 0.7			
Γ_{ν_3}(cm^{-1})					35.7 ± 0.9			
χ_{lib}					13.963 ± 0.704			
$\tilde{\nu}_{\mathrm{lib}}$(cm^{-1})					832			
$\Gamma_{\mathrm{lib}\nu_3}$(cm^{-1})					135			

Supplementary Table 2. Results of fits to data collected employing the *ppp* polarisation condition. The parameters shown in this and Table 1 are the results of a global fit to *ppp* and *ssp* spectra at all concentrations of $HClO_4$. Note that the spectral width of the 800 nm pulse was independently measured before each VSF measurement and the libration center frequency and line width were extracted from our previous work[1].

Because our principle result is the *ratio* of $\chi_{\nu_1}^{\mathrm{ssp}}/\chi_{\nu_1}^{\mathrm{ppp}}$ it is worth noting explicitly how the uncertainty we report in this ratio is calculated. Given the spectral amplitudes and their standard deviations and the covariance of these quantities we calculate the standard deviation in the resulting ratio at each bulk concentration of $HClO_4$ as:

$$\sigma_{\nu_1}^{ssp/ppp} = \left(\frac{\chi_{\nu_1}^{ssp}}{\chi_{\nu_1}^{ppp}}\right)\sqrt{\left(\frac{\sigma_{\nu_1}^{ssp}}{\chi_{\nu_1}^{ssp}}\right)^2 + \left(\frac{\sigma_{\nu_1}^{ssp}}{\chi_{\nu_1}^{ssp}}\right)^2 - 2\frac{\mathrm{cov}\left(\chi_{\nu_1}^{ssp}, \chi_{\nu_1}^{ppp}\right)}{\chi_{\nu_1}^{ssp} * \chi_{\nu_1}^{ppp}}} \tag{S1}$$

We finally note in passing that the measured signals we report are highly reproducible: when quanitatively referenced spectra collected on different days plot on top of each other. As a consequence, and as illustrated from the fit parameters, uncertainty in the $\chi_{\nu_1}^{\mathrm{ssp}}/\chi_{\nu_1}^{\mathrm{ppp}}$ ratio is dominated by uncertainty in the fit to the low signal *ppp* measurement.

The *ppp* spectra corresponding to the *ssp* spectra shown in Figure 2 in the manuscript are shown in Supplementary Figure 1. Clearly this signal is weak. However, we are interested in extracting the amplitude of the ν_1 mode subject to the constraints described above. Given these boundary conditions we find, as is hopefully clear from inspection of the data, that a nonzero *ppp* amplitude exists at all $HClO_4$ concentrations 0.2 M and above. While we globally fit all data sets to extract the center frequencies of ν_1 and ν_3 the relatively weak ν_1 amplitude, differing noise at the low frequency side of the measurement, and the coherent nature of the VSF response (leading to interference with the ν_3), leads to small frequency shifts in the apparent peak in the signal even as the data is well described by a constant resonance frequency.

$[HClO_4]$(mol/L)	$\chi^{ssp}_{\nu_1}$	$\sigma^{ssp}_{\nu_1}$	$\chi^{ppp}_{\nu_1}$	$\sigma^{ppp}_{\nu_1}$	$\mathrm{cov}\left(\chi^{ssp}_{\nu_1}, \chi^{ppp}_{\nu_1}\right)$	$\rho^{Raman}_{\nu_1}$
0	0	0	0	0	0	0
0.2	1.872	0.126	0.218	0.081	0.0212	$0.0053^{+0.0006}_{-0.0012}$
0.3	2.306	0.139	0.268	0.075	0.0206	$0.0053^{+0.0009}_{-0.0007}$
0.4	2.418	0.155	0.253	0.068	0.0216	$0.0059^{+0.0004}_{-0.0004}$
0.5	2.597	0.171	0.247	0.065	0.022	$0.0063^{+0.0003}_{-0.0003}$
0.6	2.781	0.190	0.238	0.067	0.025	$0.0068^{+0.0004}_{-0.0005}$
0.8	3.064	0.239	0.246	0.059	0.02	$0.0071^{+0.0003}_{-0.0004}$

Supplementary Table 3. Tabulated results of the global fit to the *ssp* and *ppp* data. χ_{ν_1} and the standard deviation of χ_{ν_1}, *i.e.* σ_{ν_1}, are reproduced from Tables 1 and 2. The covariance of the $\chi^{ssp}_{\nu_1}$ and $\chi^{ppp}_{\nu_1}$ amplitudes resulting from the fit: $\sigma^{ppp:ssp}_{\nu_1}$is shown in the second last column. The calculated Raman depolarisability ratio $\rho^{Raman}_{\nu_1}$ with its uncertainty is shown in the last column. The fit results for the 0.1 mol/L $HClO_4$ sample are not included in this table because, per the discussion above, we did not include the fits results from this concentration in analysis of amplitude ratios.

Supplementary Note 2. VSF Spectra of 0.6 M Perchlorate Salt Solutions

I_{sf} is plotted as a function of incident infrared frequency for an 0.6 M solution of $NaClO_4$ in Figure 2. Clearly this solution also has a distinct ν_1 mode. Comparison with the spectra plotted in Figure 1 in the manuscript suggests that the break in symmetry that makes ν_1 IR active near the interface is not the result of an interfacial change in pKa.

Supplementary Note 3. The Raman Depolarisation Ratio in Bulk and at the Interface

In the following section we develop the full description of the connection between the Raman depolarisation ratio and measured VSF intensities as described in the literature by Long and Hirose et al [2, 3] and recently reviewed by Wang et al [4]. Given a $ClO_4{}^-$ ion has $C_{3\nu}$ symmetry, assuming off diagonal terms in the polarisability tensor are small and the c-axis is taken along the rotational symmetry axis of the ion, there are three nonzero and two independent terms in the polarisability tensor: $\alpha_{aa} = \alpha_{bb}$, α_{cc}. For this molecule the Raman depolarisation ratio is defined (where $R = {}^{\alpha_{aa}}/_{\alpha_{cc}}$):

(S2) $$\rho = \frac{I_\perp}{I_\parallel} = \frac{3(\alpha_a)^2}{45(\alpha_i)^2 + 4(\alpha_a)^2} = \frac{3}{4 + 5\left[(1+2R)/(R-1)\right]^2}$$

α_i is defined,

(S3) $$\alpha_i = {}^1/_3\left(\alpha_{aa} + \alpha_{bb} + \alpha_{cc}\right)$$

and α_a is defined,

(S4)
$$(\alpha_a)^2 = {}^1/_2\left[(\alpha_{aa}-\alpha_{bb})^2 + (\alpha_{bb}-\alpha_{cc})^2 + (\alpha_{cc}-\alpha_{aa})^2 + 6(\alpha^2_{ab} + \alpha^2_{bc} + \alpha^2_{ca})\right]$$

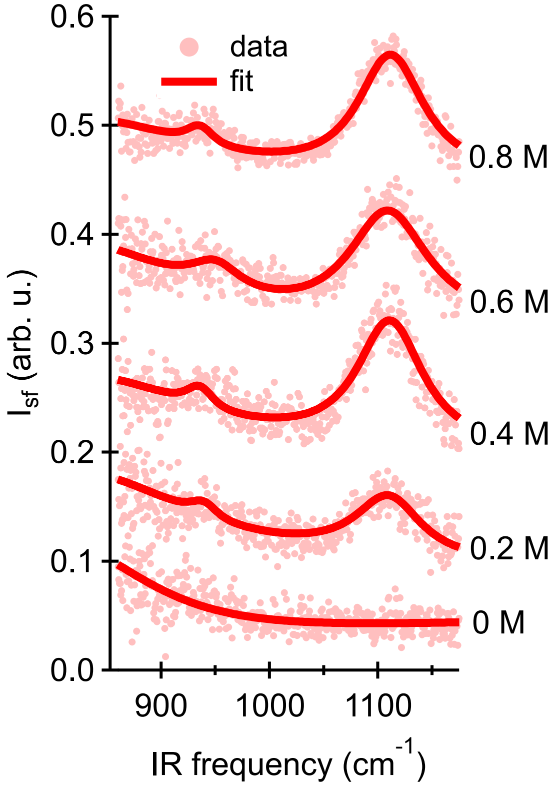

Supplementary Figure 1. VSF spectra plotted as a function of bulk concentration of $HClO_4$ collected under the *ppp* polarisation condition. Lines shown on the data are the results of global fits including the *ssp* data shown in Figure 2 in the manuscript. For clarity, only representative spectra are shown here. (Including 0.3 mol/L and 0.5 mol/L spectra will make the weak ν_1 peak hardly visible.)

Clearly, then, if we could extract R with interfacial specificity we could define an *interfacial* Raman depolarisation ratio.

As has been described extensively in the literature [5] the measured VSF intensity, *i.e.* I_{sf}, collected in reflection at the air/water interface can be written:

$$\mathrm{I}_{\mathrm{sf}} = \frac{8\pi^3 \omega_{\mathrm{sf}}^2 \sec^2 \gamma_{\mathrm{sf}}}{c^3} \left| \chi_{\mathrm{eff}}^{(2)} \right|^2 \mathrm{I}_{\mathrm{vis}} \mathrm{I}_{\mathrm{ir}} \tag{S5}$$

in which γ_i is the angle of beam i with respect to the surface normal, ω_i is the frequency of field i, c is the speed of light, I_i is the intensity of field i and $\chi_{\mathrm{eff}}^{(2)}$ is the effective, macroscopic, nonlinear susceptibility of the air/water interface. $\chi_{\mathrm{eff}}^{(2)}$ is a function of the nonlinear Fresnel factors (L_{ij}) and the polarisations of the incident and outgoing fields. These relationships can be written (assuming z is along the

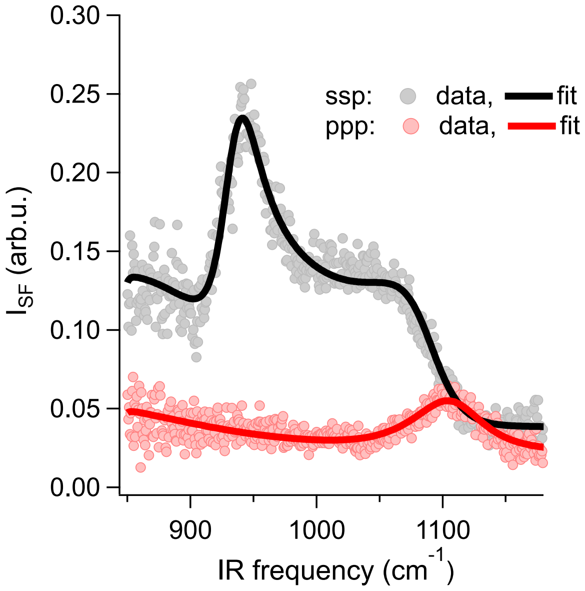

SUPPLEMENTARY FIGURE 2. I_{sf} spectrum of an 0.6 M $NaClO_4$ solution plotted as a function of IR frequency. Clearly the spectrum, and the intensity of the ν_1 mode, is quantitatively similar to that for an $HClO_4$ solution (shown in the manuscript).

surface normal and x, y the plane of the surface),

$$\chi^{(2)}_{\mathrm{eff},ssp} = L_{yy}(\omega_{\mathrm{sf}})L_{yy}(\omega_{\mathrm{vis}})L_{zz}(\omega_{\mathrm{ir}})\sin\gamma_{\mathrm{ir}}\chi^{(2)}_{yyz} \tag{S6}$$

$$\begin{aligned}\chi^{(2)}_{\mathrm{eff},ppp} = & -L_{xx}(\omega_{\mathrm{sf}})L_{xx}(\omega_{\mathrm{vis}})L_{zz}(\omega_{\mathrm{ir}})\cos\gamma_{\mathrm{sf}}\cos\gamma_{\mathrm{vis}}\sin\gamma_{\mathrm{ir}}\chi^{(2)}_{xxz} \\ & -L_{xx}(\omega_{\mathrm{sf}})L_{zz}(\omega_{\mathrm{vis}})L_{xx}(\omega_{\mathrm{ir}})\cos\gamma_{\mathrm{sf}}\sin\gamma_{\mathrm{vis}}\cos\gamma_{\mathrm{ir}}\chi^{(2)}_{xzx} \\ & +L_{zz}(\omega_{\mathrm{sf}})L_{xx}(\omega_{\mathrm{vis}})L_{xx}(\omega_{\mathrm{ir}})\sin\gamma_{\mathrm{sf}}\cos\gamma_{\mathrm{vis}}\cos\gamma_{\mathrm{ir}}\chi^{(2)}_{zxx} \\ & +L_{zz}(\omega_{\mathrm{sf}})L_{zz}(\omega_{\mathrm{vis}})L_{zz}(\omega_{\mathrm{ir}})\sin\gamma_{\mathrm{sf}}\sin\gamma_{\mathrm{vis}}\sin\gamma_{\mathrm{ir}}\chi^{(2)}_{zzz}\end{aligned} \tag{S7}$$

The nonlinear Fresnel factors are a function of the bulk and interfacial refractive indices and beam angles,

$$L_{xx}(\omega_i) = \frac{2n_{\mathrm{air}}(\omega_i)\cos\zeta_i}{n_{\mathrm{air}}(\omega_i)\cos\zeta_i + n_{\mathrm{water}}(\omega_i)\cos\gamma_i} \tag{S8}$$

$$L_{yy}(\omega_i) = \frac{2n_{\mathrm{air}}(\omega_i)\cos\gamma_i}{n_{\mathrm{air}}(\omega_i)\cos\gamma_i + n_{\mathrm{water}}(\omega_i)\cos\zeta_i} \tag{S9}$$

$$L_{zz}(\omega_i) = \frac{2n_{\mathrm{water}}(\omega_i)\cos\gamma_i}{n_{\mathrm{air}}(\omega_i)\cos\zeta_i + n_{\mathrm{water}}(\omega_i)\cos\gamma_i}\left(\frac{n_{\mathrm{air}}(\omega_i)}{n'(\omega_i)}\right)^2 \tag{S10}$$

in which ζ_i is the refracted angle of beam i (*i.e.* $n_{\mathrm{air}}(\omega_i)\sin\gamma_i = n_{\mathrm{water}}(\omega_i)\sin\zeta_i$), n_i is the, frequency dependent, refractive index of bulk phase i, and n' is the, also frequency dependent, refractive index of the interface. The material nonlinear susceptibility in the lab frame, *i.e.* $\chi^{(2)}_{ijk}$, can be expressed in terms of the nonlinear

molecular response, and the ensemble averaged orientation of ions with a $C_{3\nu}$ symmetry symmetric stretch as:

$$\text{(S11)} \quad \chi^{(2)}_{zzz} = N_s \beta^{(2)}_{ccc} \left[R\langle\cos\theta\rangle + \langle\cos^3\theta\rangle(1-R) \right]$$

$$\text{(S12)} \quad \chi^{(2)}_{xxz} = \chi^{(2)}_{yyz} = {}^1\!/\!_2 N_s \beta^{(2)}_{ccc} \left[\langle\cos\theta\rangle(1+R) - \langle\cos^3\theta\rangle(1-R) \right]$$

$$\chi^{(2)}_{xzx} = \chi^{(2)}_{yzy} = \chi^{(2)}_{zxx} = \chi^{(2)}_{zyy}$$

$$\text{(S13)} \quad = {}^1\!/\!_2 N_s \beta^{(2)}_{ccc} (1-R) \left[\langle\cos\theta\rangle - \langle\cos^3\theta\rangle \right]$$

in which $\beta^{(2)}_{abc}$ is the hyperpolarisability (*i.e.* the molecular nonlinear response), the c-axis is the rotational symmetry of the $C_{3\nu}$ molecule, θ is the orientation of the ClO_4^- with respect to the surface normal (the z-axis) and $R = \beta^{(2)}_{aac}/\beta^{(2)}_{ccc}$.

In this study we employed incident beams in the visible and infrared. These frequencies were chosen such that the infrared is resonant with Cl-O vibrations and the visible is nonresonant. Under such conditions $\beta^{(2)}$ is an anti-stokes scattering from an IR induced polarisation:

$$\text{(S14)} \quad \beta^{(2)}_{ijk} = \frac{1}{2\hbar} \frac{\alpha_{ij}\mu_k}{(\omega_n - \omega_{\text{ir}} - i\Gamma_n)}$$

in which $\hbar$ is the reduced Planck's constant, ω_n is the center frequency of the nth vibration, ω_{ir} is the frequency of the incident ir, Γ_n is the damping constant of the nth mode, α_{ij} is the polarisability tensor (as described in equations S3-S4) and μ_k is the transition dipole. Given equation S14, substituting equations S11, S12 and S13 and equations S8, S9 and S10 into equations S6 and S7 suggests that, if we know the orientation of the ClO_4^- and measure I_{sf} under the *ppp* and *ssp* polarisation conditions, the ppp/ssp ratio depends only on R. Because $\beta^{(2)}$ is a product of the polarisability and transition dipole:

$$\text{(S15)} \quad R = \frac{\beta^{(2)}_{aac}}{\beta^{(2)}_{ccc}} = \frac{\alpha_{aa} \times \mu_c}{\alpha_{cc} \times \mu_c} = \frac{\alpha_{aa}}{\alpha_{cc}}$$

Equation S15 thus implies that by taking the ratio of I_{sf} measured under the *ppp* and *ssp* polarisation conditions we can extract an R for interfacial ClO_4^- and calculate an interfacial Raman depolarisation ratio.

Supplementary Note 4. Evaluating Assumptions in the Calculation of Interfacial ρ

What is the dependence of calculated ρ on ClO_4^- orientation? Given the set of equations shown, an experimentally measured $\chi_r^{\text{ssp}}/\chi_r^{\text{ppp}}$ ratio, and assuming a ClO_4^- orientation one can extract a value of R. The resulting solution is plotted in Supplementary Figure 3. Calculated interfacial values of ρ shown in the manuscript assume the ClO_4^- anion is oriented between 0 and 45° and R values range between 0 and 1. Orientations between 45 and 90 degrees would imply ion pairing and (further) reduce ClO_4^- symmetry for which we see no spectral evidence. R values above 2 imply that the ν_1 polarisability is larger perpendicular to the Cl-O bond than parallel. Prior experiment and theory has shown that this is not the case in bulk [6]. Our electronic structure calculations suggests that this is not the case for ClO_4^- in an applied field similar in amplitude to what we would expect in the local interfacial environment.

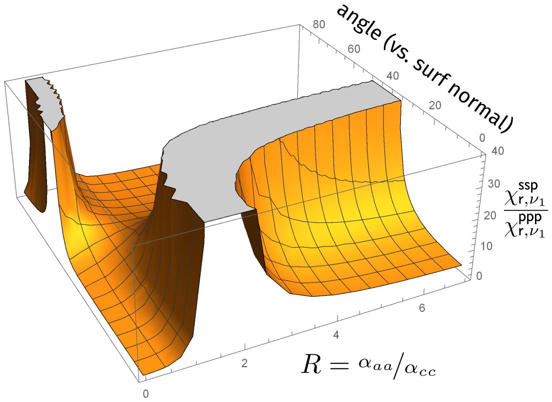

Supplementary Figure 3. Calculated relationship of the R at the IR frequencies of the ν_1 mode, measured $\chi^{ssp}_{r,\nu_1}/\chi^{ppp}_{r,\nu_1}$ and ClO_4^- orientation. The physically relevant solution to this set of equations is that with an R value between 0 and 1.

Is ClO_4^- orientation concentration dependent? The transition dipole of the ν_1 and ν_3 modes of the ClO_4^- anion are orthogonal. One consequence of this property is that changes in interfacial orientation of the ClO_4^- anion will result in changes in relative intensities of the ν_1 and ν_3 modes as a function of bulk $HClO_4$ orientation (for resonances appearing in spectra collected under a single polarisation condition). The calculated dependence of the $\chi^{ssp}_{r,\nu_3}/\chi^{ssp}_{r,\nu_1}$ are shown in Supplementary Figure 4 and the experimentally measured values, repeated from Figure 2c in the manuscript for ease of comparison, in Supplementary Figure 5. Comparison of the two figures makes clear that, if ClO_4^- orientation changes as a function of bulk concentration of $HClO_4$ (and thus presumably with increasing *interfacial* ClO_4^- concentration), this orientation change must be small.

Supplementary Note 5. Computational Details

We simulated the deformation of an isolated ClO_4^- anion exposed to external electric dipole fields along the z-axis and studied the resulting changes of the dipole moment and the related Raman depolarisation ratio. Three popular density functional approximations, *i.e.* PBEPBE[7], PBE0[8] and B3LYP[9], were employed together with a series of Gaussian-type basis sets, (aug-)cc-pVnZ with n=T, Q, 5. The calculations were performed using the GAMESS package[10]. As shown in Tables 4-11, the three methods predict a very similar influence of the external electric dipole fields on the ClO_4^- anion, with a slight method-dependent discrepancy in the calculated Cl-O bond length.

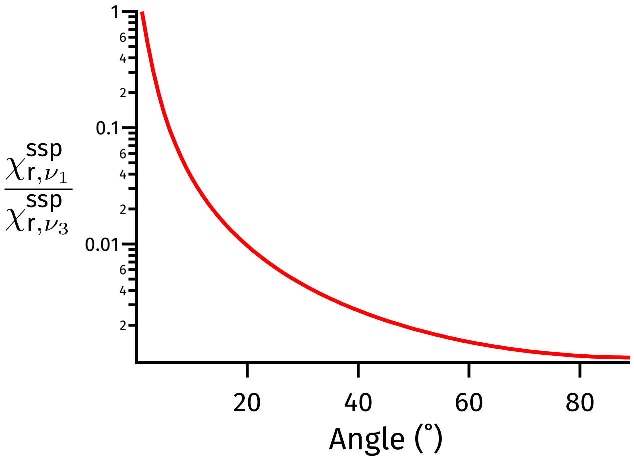

Supplementary Figure 4. Calculated $\chi^{\text{ssp}}_{\text{r},\nu_1}/\chi^{\text{ssp}}_{\text{r},\nu_3}$ ratio as a function of $ClO_4{}^-$ orientation. Clearly even few degree changes in orientation should lead to large changes in ratio.

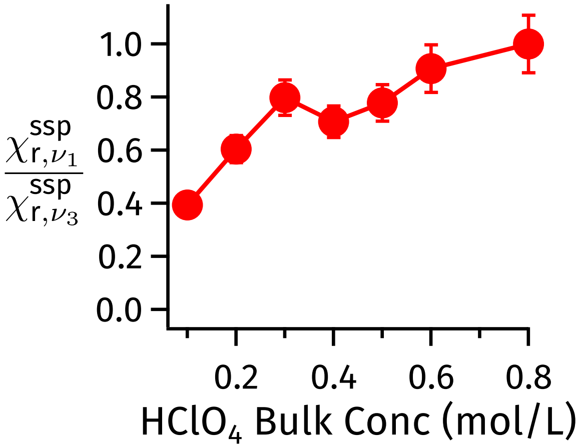

Supplementary Figure 5. Ratios of $\chi^{\text{ssp}}_{\text{r},\nu_3}/\chi^{\text{ssp}}_{\text{r},\nu_1}$ extracted from experiment for spectra collected under the *ssp* polarisation condition. The reported uncertainty is the result of propagating the error(s) in Supplementary Tables 1 and 2 as described above (when not visible error bars are less than the size of the data point). Clearly comparison of experiment with the calculated result shown in Supplementary Figure 4 suggest that $ClO_4{}^-$ orientation is relatively insensitive to bulk $HClO_4$ concentration.

E_z (Debye)	ϕ_z (meV)	Dipole	ρ	Cl-O_z (Å)	Cl-O_{other} (Å)	Angle	Cl-O_z/Cl-O_{other}	IR intens
0	0	0.0001	0.0000	1.4916	1.4916	109.47	1.00	0.0001
0.0127	136.06	-0.2316	0.0003	1.5021	1.4884	108.96	1.01	3.403
0.0254	272.11	-0.4672	0.0018	1.5138	1.4852	108.46	1.02	17.68
0.0381	408.17	-0.7089	0.0056	1.5275	1.4820	107.94	1.03	50.59
0.0508	544.23	-0.9575	0.0124	1.5434	1.4788	107.44	1.04	103.1

Supplementary Table 4. Results employing the approach described above using the PBEPBE/cc-pVTZ model chemistry.

E_z (Debye)	ϕ_z (meV)	Dipole	ρ	Cl-O_z (Å)	Cl-O_{other} (Å)	Angle	Cl-O_z/Cl-O_{other}	IR intens
0	0	0.0001	0.0000	1.4954	1.4954	109.47	1.00	0.0002
0.0127	136.06	-0.3041	0.0013	1.5074	1.4919	108.96	1.01	7.004
0.0254	272.11	-0.6242	0.0082	1.5222	1.4882	108.40	1.02	43.15
0.0381	408.17	-0.9588	0.0251	1.5397	1.4844	107.88	1.04	125.6
0.0508	544.23	-1.302	0.0450	1.5603	1.4807	107.31	1.04	229.2

Supplementary Table 5. Results employing the approach described above using the PBEPBE/aug-cc-pVTZ model chemistry.

E_z (Debye)	ϕ_z (meV)	Dipole	ρ	Cl-O_z (Å)	Cl-O_{other} (Å)	Angle	Cl-O_z/Cl-O_{other}	IR intens
0	0	0.0001	0.0000	1.4831	1.4831	109.47	1.00	0.0002
0.0127	136.06	-0.3015	0.0011	1.4946	1.4797	108.96	1.01	6.572
0.0254	272.11	-0.6160	0.0077	1.5086	1.4758	108.46	1.02	42.05
0.0381	408.17	-0.9482	0.0238	1.5248	1.4722	107.93	1.04	124.4
0.0508	544.23	-1.312	0.0417	1.5452	1.4687	107.37	1.05	238.3

Supplementary Table 6. Results employing the approach described above using the PBEPBE/aug-cc-pVQZ model chemistry.

E_z (Debye)	ϕ_z (meV)	Dipole	ρ	Cl-O_z (Å)	Cl-O_{other} (Å)	Angle	Cl-O_z/Cl-O_{other}	IR intens
0	0	0.0001	0.0000	1.4731	1.4731	109.47	1.00	0.0002
0.0127	136.06	-0.2982	0.0011	1.4845	1.4694	108.98	1.01	7.050
0.0254	272.11	-0.6076	0.0074	1.4978	1.4660	108.48	1.02	41.95
0.0381	408.17	-0.9369	0.0237	1.5137	1.4625	107.96	1.04	128.3
0.0508	544.23	-1.298	0.0392	1.5328	1.4590	107.41	1.05	249.7

Supplementary Table 7. Results employing the approach described above using the PBEPBE/aug-cc-pV5Z model chemistry.

E_z (Debye)	ϕ_z (meV)	Dipole	ρ	Cl-O_z (Å)	Cl-O_{other} (Å)	Angle	Cl-O_z/Cl-O_{other}	IR intens
0	0	0.0001	0.0000	1.4789	1.4789	109.47	1.00	0.0001
0.0127	136.06	-0.2878	0.0011	1.4909	1.4755	108.96	1.01	6.768
0.0254	272.11	-0.5818	0.0066	1.5037	1.4720	108.45	1.02	37.19
0.0381	408.17	-0.8911	0.0215	1.5195	1.4686	107.91	1.03	109.1
0.0508	544.23	-1.219	0.0437	1.5378	1.4651	107.36	1.05	204.6

Supplementary Table 8. Results employing the approach described above using the B3LYP/aug-cc-pVTZ model chemistry.

E_z (Debye)	ϕ_z (meV)	Dipole	ρ	Cl-O_z (Å)	Cl-O_{other} (Å)	Angle	Cl-O_z/Cl-O_{other}	IR intens
0	0	-0.0001	0.0000	1.4572	1.4572	109.47	1.00	0.0001
0.0127	136.06	-0.2651	0.0009	1.4680	1.4538	108.99	1.01	6.892
0.0254	272.11	-0.5390	0.0063	1.4807	1.4506	108.49	1.02	41.12
0.0381	408.17	-0.8244	0.0205	1.4951	1.4473	107.99	1.03	119.6
0.0508	544.23	-1.132	0.0415	1.5127	1.4437	107.46	1.05	230.6

Supplementary Table 9. Results employing the approach described above using the B3LYP/aug-cc-pV5Z model chemistry.

E_z (Debye)	ϕ_z (meV)	Dipole	ρ	Cl-O_z (Å)	Cl-O_{other} (Å)	Angle	Cl-O_z/Cl-O_{other}	IR intens
0	0	0.0001	0.0000	1.4620	1.4620	109.47	1.00	0.0141
0.0127	136.06	-0.2737	0.0008	1.4722	1.4589	108.98	1.01	6.315
0.0254	272.11	-0.5552	0.0054	1.4840	1.4558	108.49	1.02	36.47
0.0381	408.17	-0.8460	0.0176	1.4973	1.4528	107.99	1.03	103.4
0.0508	544.23	-1.150	0.0377	1.5124	1.4496	107.47	1.04	197.6

Supplementary Table 10. Results employing the approach described above using the PBE0/aug-cc-pVTZ model chemistry.

E_z (Debye)	ϕ_z (meV)	Dipole	ρ	Cl-O_z (Å)	Cl-O_{other} (Å)	Angle	Cl-O_z/Cl-O_{other}	IR intens
0	0	-0.0001	0.0000	1.4437	1.4437	109.47	1.00	0.0003
0.0127	136.06	-0.2538	0.0007	1.4537	1.4408	109.01	1.01	5.870
0.0254	272.11	-0.5151	0.0048	1.4651	1.4379	108.53	1.02	34.85
0.0381	408.17	-0.7857	0.0166	1.4779	1.4349	108.05	1.03	104.6
0.0508	544.23	-1.070	0.0359	1.4921	1.4316	107.56	1.04	207.6

Supplementary Table 11. Results employing the approach described above using the PBE0/aug-cc-pV5Z model chemistry.

Supplementary Note 6. Solutions with Concentrations > 1 M $HClO_4$

I_{sf} spectra of 2, 5 and 11.6 M solutions of $HClO_4$ are shown in Supplementary Figure 6. These higher concentration spectra show a clear shift in the maximum of the spectral response associated with the ν_3 mode and a gain in intensity between the ν_1 and ν_3. These trends are consistent with the splitting of ν_3 expected under conditions in which T_d symmetry is lifted. A similar gain in intensity at frequencies between the ν_1 and ν_3 modes has been previously observed in Raman spectra of aqueous $HClO_4$ solutions above 16 M and assigned to ion pairing or the appearance of molecular acid. Quantitative analysis suggests that these phenomena occur in *interfacial* ClO_4^- at concentrations more than 10× lower that in bulk.

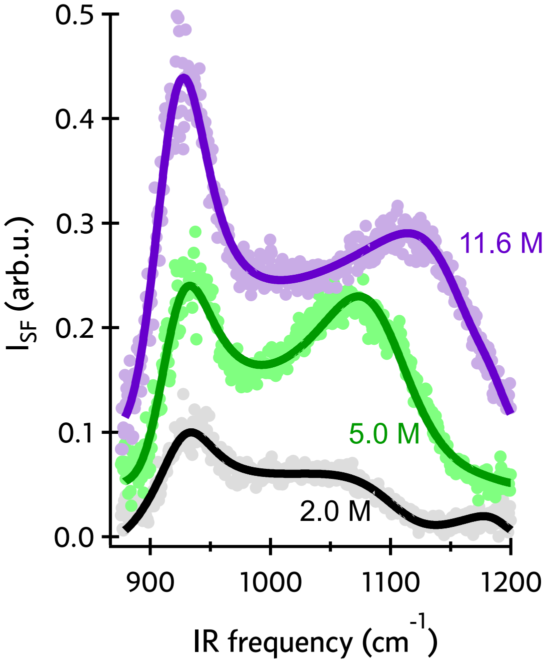

Supplementary Figure 6. VSF spectra of bulk $HClO_4$ solutions of 2, 5 and 11.6 M collected under the *ssp* polarisation condition. Dot are data, the solid lines are fits. In order to describe the data using the line shape model described above we found it necessary to introduce an additional resonance. Clearly, when comparing these data to those shown in Figure 1 in the manuscript, with increasing concentration the ν_3 mode appears to split at sufficiently high concentration. Spectra are offset for clarity.

Supplementary Note 7. Quantifying Interfacial Concentration of ClO_4^-

As discussed in the main text and above we observe a change in the anisotropy of the polarisability tensor of the ClO_4^- that is a function of bulk concentration

of $HClO_4$. We infer, particularly because $ClO_4{}^-$ is known to be have a favourable on the air/water interface, that with increasing bulk concentration from 0.1 - 1 M, interfacial concentration of the $ClO_4{}^-$ anion is also increasing. It would clearly be useful if we could quantify the interfacial concentration of $ClO_4{}^-$ over this range of bulk concentrations. In principle such quantification is possible by measurement of the change in surface tension of the acid solution with changing bulk concentration, measurement of the spectral amplitude of a single mode (rather than the ratio of spectral amplitudes we discuss in the text) in a second order nonlinear optical probe as a function of increasing bulk concentration, or from x-ray photoemission measurements.

Each of these approaches suffer significant limitations. Nevertheless we show in the following sections the constraints that surface tension and nonlinear optical measurements can offer on interfacial concentrations and discuss the, significant, limitations of both approaches. We do not further address here the application of x-ray photoemission measurements to this problem but note, as stated succinctly by Olivieri et at. recently, that actually quantifying interfacial $ClO_4{}^-$ concentrations at the air/water interface would require quantifying the inelastic mean free path of the photoelectron at this interface and the extent to which it changes as a function of anion concentration [11]. In our reading of the literature there are no clear views of either of these issues.

Interfacial Concentration through Surface Tension. Measured surface tension and calculated surface excesses as a function of bulk concentration of $HClO_4$ are shown in Supplementary Figure 7. The surface tension data are in quantitative agreement with prior studies [12, 13]. Assuming the Gibbs view of the interface – an idealized, flat, two dimensional surface, that surface tension change is solely the result of the anion, and that solute activity is equal to concentration, we calculate the interfacial concentration (*i.e.* the surface excess) following prior workers [14, 15, 16, 13] as follows.

$$\Gamma_{\mathrm{ClO_4}^-} = -\frac{1}{\mathrm{RT}}\left(\frac{\partial\gamma}{\partial\ln[\mathrm{ClO_4}^-]}\right)_{\mathrm{T,p}} \tag{S16}$$

We can approximate the derivative, $\frac{\partial\gamma}{\partial\ln[\mathrm{ClO_4}^-]} \approx \frac{\Delta\gamma}{\Delta[\mathrm{ClO_4}^-]}\cdot[\mathrm{ClO_4}^-]$. Rewriting and substituting back into equation S16 for the surface tension of $HClO_4$ at 1 M gives,

$$\begin{aligned}\Gamma_{\mathrm{ClO_4}^-} &= -\frac{1}{\left(8.3145\frac{\mathrm{kg\cdot m^2}}{\mathrm{s^2\cdot K\cdot mol}}\right)\cdot 298\ \mathrm{K}}\left(\frac{0.06925\frac{\mathrm{kg}}{\mathrm{s^2}} - 0.07196\frac{\mathrm{kg}}{\mathrm{s^2}}}{1\frac{\mathrm{mol}}{\mathrm{L}} - 0\frac{\mathrm{mol}}{\mathrm{L}}}\right)\cdot 1\frac{\mathrm{mol}}{\mathrm{L}} \\ &= 1.0937\times10^{-6}\frac{\mathrm{mol}}{\mathrm{m^2}} \end{aligned} \tag{S17}$$

At 1 M bulk concentration density of $ClO_4{}^-$ per square nanometer can then be calculated,

$$\begin{aligned} A_{\mathrm{ClO_4}^-} &= 1.0937\times10^{-6}\frac{\mathrm{mol}}{\mathrm{m^2}}\cdot\left(\frac{\mathrm{m^2}}{10^{18}\ \mathrm{nm^2}}\right)\cdot\left(\frac{6.022\times10^{23}}{\mathrm{mol}}\right) \\ &= 0.659\ \mathrm{nm^{-2}} \end{aligned} \tag{S18}$$

This result implies a radius of the adsorbed $ClO_4{}^-$, presumably including its solvation shell, of 6.6 Å.

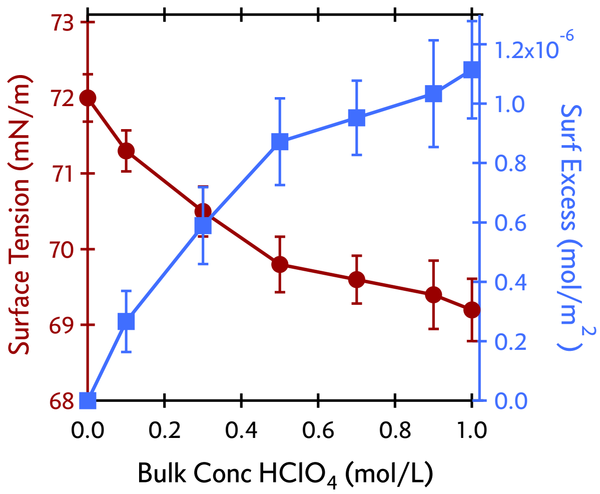

Supplementary Figure 7. Measured surface tension and calculated surface excess of the air/aqueous solution interface as a function of bulk concentration of $HClO_4$. The reported surface tensions are the average and standard deviation of twenty measurements at each concentration. Uncertainty in surface excess is the result of propagating the uncertainty in surface tension.

Interfacial Coverage through Nonlinear Optics. In the manuscript we focus on the physical understanding of the ratio of the extracted spectral amplitudes ($\chi^{\text{ssp}}_{r,\nu_1}/\chi^{\text{ppp}}_{r,\nu_1}$). As described in the manuscript and above in the Supporting Information this quantity is independent of interfacial population. However, the spectral amplitude of the ν_1 mode collected under the *ssp* polarisation condition is not. Plotting the integrated resonance amplitude, normalizing to the signal at 0.8 M $HClO_4$, as a function of bulk concentration we are left with the results shown in Supplementary Figure 8. Fitting this data with a Langmuir isotherm derived from a reaction in which the adsorbing ${ClO_4}^-$ anion replaces a water molecules, *i.e.* ClO_4^-(aq) + H_2O-surf $\rightleftharpoons$ H_2O(aq) + ClO_4^--surf, suggests the ${ClO_4}^-$ anion has a ΔG_{ads} at the air/water interface of -15 $\frac{\text{kJ}}{\text{mol}}$. Theoretical estimates of the ΔG_{ads} of the anion in the limit of infinite dilution range from -6.3 to -12.6 $\frac{\text{kJ}}{\text{mol}}$ [17]. We have no independent insight that the adsorption of the ${ClO_4}^-$ ion at the air/water interface is best understood as displacing a single water molecule and thus other, equally valid, adsorption models are possible. Such models may lead to different ΔG_{ads} inferred from the same data. For example, if we construct a Langmuir isotherm from an adsorption reaction in which no water is present, *i.e.* ${ClO_4}^-$(aq) + surf $\rightleftharpoons$ ClO_4^--surf, we find ΔG_{ads}= -5.1$\frac{\text{kJ}}{\text{mol}}$. In any case, regardless of the adsorption model employed (and the resulting calculated ΔG_{ads} of ${ClO_4}^-$ from this data) plotting the $\chi^{\text{ssp}}_{r,\nu_1}$ as a function of bulk concentration in this manner makes clear that in the range of bulk concentrations we interrogate in this paper we go from 0.5 - 1 monolayer of surface coverage.

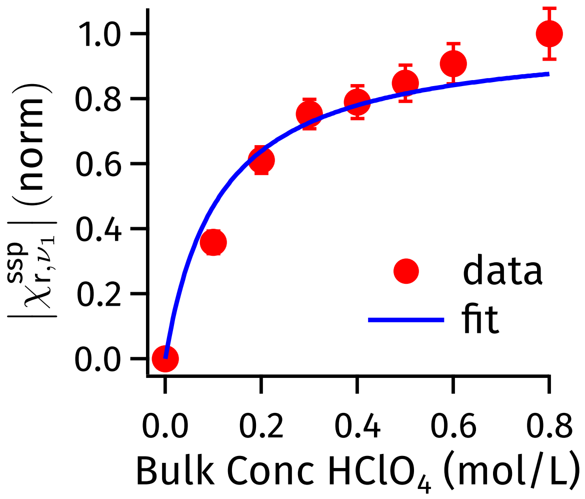

Supplementary Figure 8. The red dots are the spectral amplitude of the ν_1 mode normalized to the amplitude at 0.8 mol/L bulk concentration of $HClO_4$ and plotted as a function of bulk concentration. Because the line width of the ν_1 mode is concentration independent over this range, this quantity is proportional to interfacial population and thus the normalized quantity equivalent to surface coverage (assuming 0.8 mol/L bulk concentration reflects surface saturation). Uncertainty shown is from the fit to the data (when not shown it is smaller than the points) and is propagated from the results in Table 1. Solid blue line is a fit of the Langmuir model based on the water displacement reaction to this data that results in an inferred ΔG_{ads} of -15.5 $\frac{kJ}{mol}$. Both Langmuir models discussed in the text fit the data equally well.

It is important to note that one result of the results described in this manuscript is that this analysis ***cannot*** be quantitatively correct. This approach assumes that the nonlinear optical response per molecule, *i.e.* the hyperpolarisability $\beta^{(2)}$, is independent of interfacial concentration. We have demonstrated in this work that, at least for the Cl-O vibrations of the ClO_4^- anion this is not the case and that this assumption should only be made with ***great*** care for any SHG/VSF studies. However, because our calculations illustrate that the sense of the interface induced changes is to *increase* both the IR transition dipole and polarisability (we approximate the polarisability by taking the derivative of the dipole moment with respect to the applied field). This suggests that the changes in the single molecule response act to make our adsorption isotherm appear artificially steep and that thus the adsorption energy we extract and the lower bound of surface coverages we extract from the Langmuir isotherm are upper limits.

Supplementary References

[1] Tong, Y., Kampfrath, T. & Campen, R. K. Experimentally Probing the Libration of Interfacial Water: the Rotational Potential of Water is Stiffer at the Air/Water Interface than in Bulk Liquid. *Phys Chem Chem Phys* **18**, 18424–18430 (2016).

[2] Long, D. A. *The Raman Effect: A Unified Treatment of the Theory of Raman Scattering by Molecules* (Wiley, 2001).

[3] Hirose, C., Akamatsu, N. & Domen, K. Formulas for the Analysis of Surface Sum-Frequency Generation Spectrum by CH Stretching Modes of Methyl and Methylene Groups. *J Chem Phys* **95**, 997–1004 (1992).

[4] Wang, H., Gan, W., Lu, R., Rao, Y. & Wu, B.-H. Quantitative Spectral and Orientational Analysis in Surface Sum Frequency Generation Vibrational Spectroscopy (SFG-VS). *Int Rev Phys Chem* **24**, 191–256 (2005).

[5] Lambert, A. G., Davies, P. B. & Neivandt, D. J. Implementing the Theory of Sum Frequency Generation Vibrational Spectroscopy: A Tutorial Review. *Appl Spect Rev* **40**, 103–145 (2005).

[6] Hyodo, S.-a. Depolarization of the ν_1 Raman Band of ClO_4^- in $LiClO_4$/Ethylene Carbonate-Water Solution. *Chem Phys Lett* **161**, 245–248 (1989).

[7] Perdew, J., Burke, K. & Ernzerhof, M. Generalized Gradient Approximation Made Simple. *Phys Rev Lett* (1996).

[8] Perdew, J. P., Ernzerhof, M. & Burke, K. Rationale for Mixing Exact Exchange with Density Functional Approximations. *J Chem Phys* **105**, 9982–9985 (1996).

[9] Becke, A. D. Density Functional Thermochemistry. III. The Role of Exact Exchange. *J Chem Phys* **98**, 5648–5652 (1993).

[10] Schmidt, M. W. *et al.* General Atomic and Molecular Electronic Structure System. *J Comput Chem* **14**, 1347–1363 (1993).

[11] Olivieri, G. *et al.* Quantitative Interpretation of Molecular Dynamics Simulations for X-ray Photoelectron Spectroscopy of Aqueous Solutions. *J Chem Phys* **144**, 154704 (2016).

[12] Neros, C. A. & Eversole, W. G. The Surface Tension of Aqueous Perchloric Acid at 15°, 25°, and 50° c. *J Phys Chem* **45**, 388–395 (1941).

[13] Drzymala, J. & Lyklema, J. Surface Tension of Aqueous Electrolyte Solutions. Thermodynamics. *J Phys Chem A* **116**, 6465–6472 (2011).

[14] Onsager, L. & Samaras, N. N. T. The Surface Tension of Debye-Hückel Electrolytes. *J Chem Phys* **2**, 528–536 (1934).

[15] Randles, J. E. B. Structure at the free surface of water and aqueous electrolyte solutions, physics and chemistry of liquids. *Phys Chem Liq* **7**, 107–179 (1977).

[16] Petersen, P. B. & Saykally, R. J. On the Nature of Ions at the Liquid Water Surface. *Ann Rev Phys Chem* **57**, 333–364 (2006).

[17] Baer, M. D., Kuo, I.-F. W., Bluhm, H. & Ghosal, S. Interfacial Behaviour of Perchlorate Versus Chloride Ions in Aqueous Solutions. *J Phys Chem B* **113**, 15843–15850 (2009).
